# Empower: Design of a digital intervention for workplace stress and mental health. A European study

**DOI:** 10.1192/j.eurpsy.2023.393

**Published:** 2023-07-19

**Authors:** C. M. Van Der Feltz-Cornelis, J. Shepherd, B. Olaya, C. Vanroelen, J. Gevaert, O. Borrega Cepa, R. M. Bernard, D. Merecz-Kot, M. Sinokki, L. Hakkaart-van Roijen, J. M. Haro, C. EMPOWER

**Affiliations:** ^1^Health Sciences, University of York, York, United Kingdom; ^2^Innovation and Teaching Unit, Parc Sanitari Sant Joan de Déu, CIBERSAM, Sant Boi de Llobregat, Spain; ^3^Interface Demography, Vrije Universiteit Brussel, Brussels, Belgium; ^4^Òmada Interactiva, SLL, Barcelona, Spain; ^5^Swiss Paraplegic Research, Nottwil, Switzerland; ^6^Institute of Psychology, University of Lodz, Lodz, Poland; ^7^Turku Centre for Occupational Health, University of Turku, Turku, Finland; ^8^Erasmus School of Health Policy and Management (ESHPM), Erasmus University Rotterdam, Rotterdam, Netherlands; ^9^Innovation and Teaching Unit, Parc Sanitari Sant Joan de Déu, CIBERSAM, Barcelona, Spain

## Abstract

**Introduction:**

Work stress, anxiety and depression have an enormous impact on the well-being of employees, their employers, and society. Due to the loss of productivity, common mental disorders have a substantial economic impact. Major depression alone has been attributed to 50% of long-term absences from work, and depressive symptoms are related to lowered productivity while at work. Anxiety also contributes to loss of productivity and sickness absence. Treatment of common mental disorders in a work setting may improve symptoms, however, that does not automatically lead to improved work productivity. Addressing mental well-being at the workplace might improve work functioning, and digital interventions have been introduced with that objective. However, their evaluation in research has been limited.

The European Intervention to Promote Wellbeing and Health in the Workplace (EMPOWER) digital intervention is designed to provide and evaluate an integrative user programme that meets the needs of employees and employers in addressing work stress.

This work was supported by the European Union Horizon 2020 Research and Innovation Programme Health (grant number APP1195937, 848180). The EMPOWER project started 1.1.2020 and is currently ongoing.

**Objectives:**

We aim to

1) describe the design and development of the digital intervention.

2) culturally validate the intervention in three countries

3) test the prototype and beta version for its usability in the RCT to evaluate its effect in four countries that is currently ongoing.

**Methods:**

A user-centred design process was followed from January 2020 until November 2021 to create a beta version for usability testing. A tailored algorithm was developed to provide support at the individual employee level and the company level. Each element of the digital intervention was translated and culturally validated in four languages in Spain, the United Kingdom, Poland, and Finland. Usability testing was conducted in each country (n=31) to explore validity, usability, and user experience.

**Results:**

The digital intervention consists of a website and a mobile application (app). The website has a public section and an employer portal that provides recommendations to reduce psychosocial risks in their company based upon clustered input from employees. The app provides algorithm-based personalised content after assessing a user’s physical and psychological symptoms, work functioning, and psychosocial risk factors for work stress. The usability testing improved the flow through the app and high ease of use and completion of tasks by participants.

**Conclusions:**

The EMPOWER digital intervention is a tailored multimodal intervention addressing wellbeing, work stress, mental and physical health problems, and work productivity. Usability testing provided validation of the app as version to be evaluated in the EMPOWER RCT.

**Disclosure of Interest:**

None Declared

